# Chloroplast genomes of nine *Primula* species: comparative genomics and phylogenetic implications

**DOI:** 10.3389/fpls.2026.1889564

**Published:** 2026-09-16

**Authors:** Yanru Zhang, Shihao Jiang, Qinyi Wang, Xingyu Du, Jiazhen Wu, Xiang Li, Jieli Yue, Min Pan, Xiaoqin Liu, Yizhen Shao, Xianhua Gu, Liang Dou, Jing Zhou, Zhixi Fu

**Affiliations:** 1 College of Life Sciences, Sichuan Normal University, Chengdu, China; 2 Meishan Ecological Environment Monitoring Center Station, Meishan, China; 3 College of Life Science, Henan Agricultural University, Zhengzhou, China; 4 Sichuan Leshan Ecological Environment Monitoring Center Station, Leshan, China; 5 Museum of Natural History, College of Life Sciences, Sichuan University, Chengdu, China; 6 Sustainable Development Research Center of Resources and Environment of Western Sichuan, Sichuan Normal University, Chengdu, China

**Keywords:** ancestral state reconstruction, chloroplast genome, comparative analysis, divergence time, phylogenetic analysis, primula

## Abstract

**Introduction:**

*Primula* is the largest genus in Primulaceae and a classic model for studying heterostyly and plant evolution.

**Methods:**

In this study, we sequenced, assembled, and annotated the complete chloroplast genomes of seven *Primula* species from China. Two additional plastomes of *Primula dejuniana* and *Primula meishanensis*, previously generated and published by our research group, were retrieved from GenBank and integrated into our dataset. We further combined these nine plastome resources with 48 published *Primula* plastomes and three outgroup plastomes downloaded from GenBank, forming a total dataset of 60 chloroplast genomes to conduct comparative genomic analyses, repeat sequence detection, hypervariable region identification, phylogenetic reconstruction, divergence time estimation, and ancestral trait reconstruction.

**Results:**

The chloroplast genomes exhibited a typical quadripartite structure and high conservation, with lengths ranging from 151, 555 to 154, 653 bp. We detected 433 SSRs, 242 long repeat sequences, and six highly variable regions that can be used as candidate molecular markers. Phylogenetic analyses firmly clarified the relationships of several sections and newly sequenced species. Divergence time analyses indicated that *Primula* initiated its rapid radiation at approximately 19.59 Mya (95% HPD: 13.19–25.89 Mya, Early Miocene). Ancestral state reconstruction supported that the genus was ancestrally characterized by umbellate inflorescences, heterostyly, purple corollas, and campanulate calyces, with derived traits evolving repeatedly.

**Discussion:**

Our results provide valuable chloroplast genomic resources and robust phylogenetic evidence for the evolutionary study of *Primula* species.

## 1 Introduction

As the largest genus within Primulaceae, *Primula* encompasses approximately 500 species classified into 38 sections (Hu and Kelso, 1996; Richards, 2002; Li et al., 2024). Over two-thirds of this remarkable diversity is concentrated in the mountains of southwestern China (Hu and Kelso, 1996; Yan et al., 2010). These herbaceous perennials are characterized by a basal rosette of leaves and a distinctive naked scape bearing flowers typically arranged in umbels (Li et al., 2023b; Hu and Kelso, 1996; Richards, 2002). Beyond its global horticultural appeal, the genus serves as a classic model system for investigating heterostyly and the evolution of self-incompatibility mechanisms (Ren et al., 2018; Yan et al., 2010, 2025; Huu et al., 2020). The taxonomy of *Primula* has undergone multiple revisions based on morphological characteristics. Morphological classifications have been successively refined since Smith & Fletcher’s 31-section system (Smith and Fletcher, 1947); later authors proposed seven (Wendelbo, 1961) or six subgenera (Richards, 1993) to accommodate putative reticulate evolution. Hu and Kelso (1996) categorized Chinese *Primula* species into 24 sections (Hu and Kelso, 1996).

In recent years, complete plastid genome sequencing has become a cornerstone of plant systematics, providing unprecedented resolution for inferring phylogenetic relationships and delimiting taxonomic boundaries (Neuhaus and Emes, 2000; Liang et al., 2020; Song et al., 2024; Xie et al., 2025; Zhao et al., 2023). Chloroplasts are multifunctional organelles whose roles extend beyond photosynthesis to encompass critical metabolic, developmental, and stress-responsive signaling networks (Zhang et al., 2024; Wang et al., 2024b; Medina-Puche et al., 2020; Llorente et al., 2020). Their genomes—circular molecules typically ranging from 100 to 200 kb in higher plants—exhibit a highly conserved quadripartite structure: two inverted repeats (IRs) separate the large and small single-copy regions (LSC and SSC), with ribosomal RNA genes residing within the IRs (Downie and Palmer, 1992; Zheng et al., 2016; Guo et al., 2021; Kim et al., 2016). This architectural conservation, combined with sufficient sequence variation, uniparental inheritance, and moderate mutation rates, renders chloroplast genomes powerful tools for phylogenetic inference and species identification (Moore et al., 2007; Sugiura, 1992; He et al., 2019; Ran et al., 2020). Indeed, whole plastomes and protein-coding datasets have been shown to provide superior resolution compared to traditional markers, leading to their widespread adoption as ‘super-barcodes’ in plant systematics (Xia et al., 2022; Miao et al., 2017; Wang et al., 2024a; Shaw et al., 2014).

With the rapid development of sequencing techniques, both enriched and unenriched DNA libraries can now be sequenced using efficient sequencing methods such as sequencing-by-synthesis (e.g., Illumina) or single‐molecule real‐time sequencing (SMRT; e.g., PacBio) (Wicke and Schneeweiss, 2015). The chloroplast DNA genome in plants with complex genomes has been demonstrated to be relatively cost-effective and readily amenable to sequencing (Guo et al., 2017; Saarela et al., 2018).

In this study, we obtained plastid genomes for nine Chinese *Primula* species via Illumina paired-end sequencing resources. Seven species, namely *P. wawushanica* G. Hao, C. M. Hu & Yuan Xu, *P. secundiflora* Franch., *P. gemmifera* Batalin, *P. flava* Maxim., *P. fangii* F. H. Chen & C. M. Hu, *P. polyneura* Franch. and *P. purdomii* Craib were newly sequenced, assembled and annotated in the present study. The plastomes of *Primula meishanensis* K.Huang & Z.X.Fu and *Primula dejuniana* G.Hao, C.M.Hu & Yuan Xu were retrieved from our previously published dataset (Li et al., 2024). All nine plastome resources analyzed herein represent *Primula* species endemic to southwest Sichuan, the global diversity center of this genus. To date, these nine endemic species have never been subjected to integrated plastid genomic research as a collective group, leaving their phylogenetic affinities largely uncharacterized, and our newly obtained genomic resources therefore fill this untapped research gap for these taxa. A general characteristic analysis of cp genomes was performed for the nine *Primula* species sequenced in this study. In addition, repeated sequence analysis, genome structure analysis, comparative analysis, and phylogenetic analysis were conducted on the nine *Primula* species in this study and 49 cp genomes of *Primula* were submitted to the GeneBank database. By generating nine complete chloroplast genomes of Chinese *Primula*, we aimed to: (1) elucidate the complete cp genome sequences for nine *Primula* species; (2) conduct a comparative analysis of cp genome structures within the genus; (3) characterize SSR and repetitive-sequence variation; (4) identify hyper-mutable regions and potential marker genes; (5) reconstruct robust phylogenetic relationships within *Primula*; and (6) perform ancestral-state reconstruction of key morphological traits (inflorescence architecture, heterostyly, corolla color, calyx shape) to trace their evolutionary origins and transitions across the genus.

## 2 Materials and methods

### 2.1 Sample collection and sequencing

In 2024, nine *Primula* species were collected during routine field monitoring under permits issued by the relevant local and national authorities. Seven of these species were newly sequenced in the present study. All newly sequenced materials were taxonomically verified by the corresponding author, and voucher specimens were deposited in the Herbarium of Sichuan Normal University (SCNU; Index Herbariorum code: SCNU), a publicly accessible herbarium registered in the Index Herbariorum. Detailed voucher information is provided in the “Ethics approval and consent to participate” section. Genomic DNA was isolated from leaf tissue following a standard CTAB protocol (Doyle, 1987), after which paired-end sequencing libraries were constructed according to the Illumina DNA Library Construction Guide (Allen et al., 2006). The complete cp genomes were sequenced on the Illumina HiSeq XTen platform (San Diego, CA, USA), and the complete data were obtained after quality control. The raw sequencing reads generated in this study have been deposited in the Genome Sequence Archive (GSA) at the National Genomics Data Center, China National Center for Bioinformation, under accession number CRA039956 (https://ngdc.cncb.ac.cn/gsa) (Zhang et al., 2025, Members and Partners, 2024).

The chloroplast genomes of *P. dejuniana* (PQ213817) and *P. meishanensis* (PQ213816) were recently made available in GenBank following their initial assembly and annotation in our laboratory (Li et al., 2024). It is important to note that these sequences have not previously been subjected to the comprehensive comparative genomic, phylogenomic, and ancestral trait reconstruction analyses presented in the current study.

Additionally, we downloaded 48 published species of *Primula* and 3 species as outgroups (*Ardisia polysticta*, *Androsace bulleyana* and *Androsace erecta*) from the GenBank database, totaling 60 samples (**Supplementary Table 1**).

### 2.2 Chloroplast genome assembly and annotation

Complete plastomes were assembled *de novo* with GetOrganelle v1.7.7.1 (Jin et al., 2020), and assembly graphs were visually inspected for accuracy and completeness using Bandage v.0.8.1 (Wick et al., 2015). CPGAVAS2 was employed to annotate the genomes with manual checking and modification. The physical maps of the cp genomes were generated using OGDRAW (v1.3.1) (Greiner et al., 2019) and then manually edited. The final annotated genomes were submitted to NCBI GenBank, obtaining the accession numbers. Annotated sequences were deposited in NCBI GenBank under the accession numbers listed in **Supplementary Table 1**.

### 2.3 Codon usage analysis

All protein-coding sequences (CDS) extracted from the plastomes of nine *Primula* species were subjected to codon usage bias analysis. Two tools were applied to calculate multiple relevant indices: CodonW software (http://codonw.sourceforge.net/) and the online EMBOSS CUSP tool (http://imed.med.ucm.es/EMBOSS). The measured indicators included relative synonymous codon usage (RSCU), effective number of codons (ENC), total GC content (GCall), GC content at the first, second and third codon positions (GC1, GC2, GC3), and GC content of synonymous third codon positions (GC3s). RSCU values greater than 1 represent preferred codons, while values below 1 indicate rarely used codons (Sharp et al., 1986).

To explore the evolutionary forces shaping codon bias, we further constructed an ENC plot and a neutrality plot. For the ENC plot, ENC values of all CDS were plotted against GC3s, alongside a theoretical standard curve generated solely under mutational pressure based on the formula: ENC = 2+GC3s+29/[GC3s²+(1−GC3s) ²] (Wright, 1990). The degree of deviation of data points from this curve was used to assess the joint effects of mutation and natural selection. For the neutrality plot, GC3 was set as the x-axis and the average of GC1 and GC2 (GC12) as the y-axis; linear correlation analysis was then conducted to compare base composition variation across three codon positions and quantify the relative magnitudes of mutational and selective pressures (Wei et al., 2014; Wen et al., 2017).

### 2.4 Repetitive sequences in chloroplast genome analysis

Simple sequence repeats (SSRs) were identified with MISA (Beier et al., 2017) (https://webblast.ipk-gatersleben.de/misa/index.php) using the following minimum thresholds: mono-nucleotides ≥ 10, di- ≥ 5, tri- ≥ 4, tetra- ≥ 3, penta- ≥ 3, and hexa-nucleotides ≥ 3, with intervening sequences ≥ 100 bp. Dispersed repeats were detected with REPuter (Kurtz et al., 2001) (https://bibiserv.cebitec.uni-bielefeld.de/reputer) under the constraints of ≥ 30 bp length, ≥ 90% sequence identity, and Hamming distance ≤ 3. Both analyses were performed on the nine *Primula* plastomes.

### 2.5 Comparative analysis of IR regions

IRscope (https://irscope.shinyapps.io/irapp/) was employed to visualize and compare the junctions among the LSC, IR, and SSC regions of the chloroplast genomes (Amiryousefi et al., 2018).

### 2.6 Sequence divergence analysis

Genome-wide sequence divergence among the nine *Primula* plastomes was assessed using mVISTA (https://genome.lbl.gov/vista/mvista/submit.shtml) with the Shuffle-LAGAN alignment model, employing *Ardisia polysticta* as the reference (Frazer et al., 2004). Compare the non-coding and coding regions of the exon, intron, and chloroplast genomes.

### 2.7 Highly variable area segments analysis

To detect mutational hotspots, we computed nucleotide diversity (π) across the aligned plastomes using a 100 bp sliding window with 25 bp increments in DnaSP v5 (Librado and Rozas, 2009).

### 2.8 Phylogenetic analysis

We generated three phylogenies from the identical CDS alignment matrix of plastid protein-coding sequences. IQ-TREE software was used to perform ML phylogenetic inference (**Supplementary Figure 1**), with the optimal substitution model automatically selected by ModelFinder embedded in IQ-TREE (Nguyen et al., 2015). MrBayes v 3.2.6 was adopted to generate Bayesian topological tree (**Supplementary Figure 2**) (Ronquist et al., 2012), serving as supplementary verification for topological consistency with the ML tree. Divergence time estimation of *Primula* was performed using BEAST v.2.7.3 (Bouckaert et al., 2019) with two fossil calibration points following a normal distribution (delta = 5.1). The stem node separating *Primula* and *Androsace* was constrained to 32 ± 10 Mya (de Vos et al., 2014), and the crown node of *Primula riosiae* was set to 15.97 ± 10 Mya based on previously reported seed fossil evidence (Czaja and Alexander, 2003). An optimised relaxed clock and Yule prior were applied, with the MCMC chain run for 100 million generations and sampled every 1000 steps. Tracer was used to assess MCMC convergence, with all ESS values required to exceed 200. TreeAnnotator v2.7.3 was used to summarize the maximum clade credibility (MCC) chronogram, discarding the first 10% samples as burnin; the final chronogram was visualized using Chiplot (https://www.chiplot.online/) (Xie et al., 2023a). The time-calibrated MCC tree was used for subsequent ancestral state reconstruction, while the ML and BI trees served as topological references.

### 2.9 Ancestral character state reconstructions

Four key morphological traits (inflorescence type, corolla colour, style, and calyx shape) were selected for ancestral state reconstruction. These traits are taxonomically diagnostic and phylogenetically informative for *Primula*; heterostyly represents the iconic reproductive system of the genus, corolla colour is closely related to pollinator adaptation and environmental stress tolerance, and inflorescence and calyx morphology are relatively conserved, well-documented in floras and herbarium records, and thus suitable for reliable evolutionary inference. The character data used for ancestral traits reconstruction were obtained from Flora of China (https://www.iplant.cn/foc). The character state codes associated with these data include (a) calyx: (A) campanulate, (B) narrowly campanulate, (C) unknown; (b) corolla color: (A) purple, (B) yellow, (C) white, (D) unknown; (c) inflorescence: (A) umbel, (B) capitulum, (C) spike, (D) receme; (d) style: (A) heterostyly, (B)homostyly, (C) co-occurrence of hetero- and homostyly, (D) unknown (**Supplementary Table 5**).

Based on the resolved phylogenetic topology, ancestral state reconstruction of the four discrete morphological traits was performed using RASP v4.3 (Yu et al., 2015). Three Markov k-state (Mk) evolutionary models, namely the equal-rate (ER), symmetric-rate (SYM), and all-rates-different (ARD) models, were independently fitted for each trait. For the inflorescence trait, the character states C and D were each represented by only a single species, resulting in insufficient cross-state transition information across the phylogenetic tree. Such limited sampling prevented stable estimation of all free rate parameters of the ARD model and increased the risk of numerical anomalies and model overfitting. Therefore, states C and D were merged into state B prior to subsequent analyses. The remaining three morphological traits exhibited balanced taxonomic sampling across all character states and were analyzed based on their original coding schemes. The optimal evolutionary model for each trait was selected according to the log-likelihood values. Ancestral state probabilities were visualized on the time-calibrated maximum clade credibility (MCC) tree based on the best-fitting ARD model, while the reconstruction results derived from the ER and SYM models were presented in **Supplementary Figures 4**, **5**, respectively.

## 3 Results

### 3.1 General features of the *Primula* chloroplast genome

The cp genomes of nine *Primula* species were sequenced and analyzed, revealing a range of genome lengths from 151, 555 bp in *P. secundiflora* to 154, 653 bp in *P. gemmifera* (**Table 1**). These genomes are typical circular DNA molecules, consistent with the structure of most angiosperm cp genomes. The GC content of these genomes ranged from 36.85% in *P. gemmifera* to 37.08% in *P. polyneura*, aligning with previously published data for *Primula* cp genomes (**Figure 1**). The cp genomes of the *Primula* species exhibit a typical four-part structure, consisting of the large single-copy (LSC) region, the small single-copy (SSC) region, and two inverted repeat (IR) regions. The LSC region ranged from 82, 928 bp in *P. wawushanica* to 85, 201 bp in *P. gemmifera*, while the SSC region ranged from 17, 730 bp in *P. meishanensis* to 17, 826 bp in *P. secundiflora*. The IR region ranged from 50, 350 bp in *P. dejuniana* to 51, 682 bp in *P. flava*. As is typical in angiosperm plastomes, the IR regions exhibited elevated GC levels relative to the single-copy regions. (**Table 1**). The number of genes in the cp genomes varied slightly among the species, ranging from 128 in *P. purdomii* and *P. secundiflora* to 133 in *P. meishanensis*. These genes include 84 to 88 protein-coding genes, 35 to 37 tRNA genes, and 8 rRNA genes. The proteins encoded by these genes are involved in photosynthesis, self-replication, and others, among which unknown proteins are also present (**Table 2**). Of these, seventeen were intron-containing genes, and fourteen contained an intron (*ndhA*, *ndhB*, *petB*, *petD*, *atpF*, *rpl16*, *rpl2*, *rps16*, *rpoC1*, *trnA-UGC*, *trnG-UCC*, *trnK-UUU*, *trnL-UAA*, *trnV-UAC*), and three contained two introns (*rps12*, *clpP*, *pafI*). The cp genomes of the nine *Primula* species exhibit a conserved structure and gene content, with minor variations in genome length and gene number. The genes are involved in essential functions such as photosynthesis, self-replication, and other metabolic processes. The presence of introns and multi-copy genes is consistent with the genomic structure observed in other angiosperms. These findings provide valuable insights into the evolutionary relationships and functional genomics of the *Primula* genus.

**Table 1:** Summaries of complete cp genomes of nine *Primula* species.

Species	Total length (bp)	LSC (bp)	IR (bp)	SSC (bp)	Total GC content (%)	Total genes	Number of CDS genes	Number of tRNA genes	Number of rRNA genes
*P. fangii*	154, 286	84, 850	51, 656	17, 780	37.01	130	86	36	8
*P. purdomii*	151, 559	83, 242	50, 494	17, 823	36.94	128	85	35	8
*P. polyneura*	153, 453	84, 415	51, 270	17, 768	37.08	130	86	36	8
*P. flava*	154, 347	84, 915	51, 682	17, 750	36.97	130	86	36	8
*P. gemmifera*	154, 653	85, 201	51, 664	17, 788	36.85	131	86	37	8
*P. secundiflora*	151, 555	83, 235	50, 494	17, 826	36.94	128	84	36	8
*P. wawushanica*	152, 999	82, 928	52, 293	17, 778	37.00	130	85	37	8
*P. meishanensis*	151, 988	83, 888	50, 370	17, 730	37.01	133	88	37	8
*P. dejuniana*	152, 175	84, 052	50, 350	17, 773	36.93	131	86	37	8

IR (bp) indicates the total length of both inverted repeat regions (IRa + IRb).

**Figure 1:**
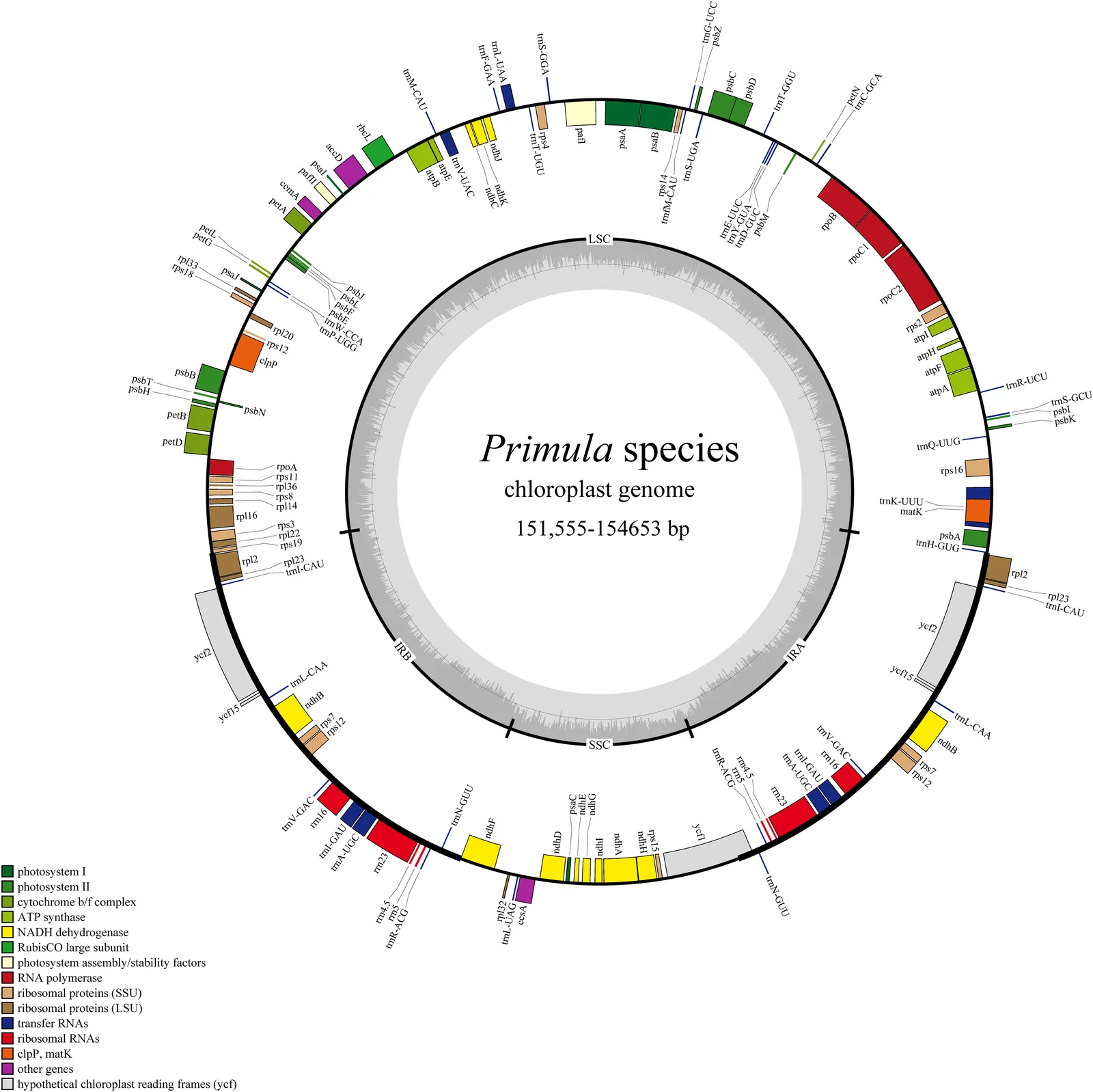
Gene map of the complete cp genomes of *Primula* species. Genes inside the circle are transcribed clockwise, and those on the outside are transcribed counter-clockwise. Genes belonging to different functional groups have been color-coded. The darker grey area in the inner circle corresponds to GC content, whereas the lighter grey corresponds to AT content.

**Table 2:** Genes in the P. fangii cp genomes.

Category	Gene group	Gene name
Photosynthesis	Subunits of photosystem I	*psaA*, *psaB*, *psaC*, *psaI*, *psaJ*
	Subunits of photosystem II	*psbA, psbB, psbC, psbD, psbE, psbF, psbH, psbI, psbJ, psbK, psbL, psbM, psbN, psbT, psbZ*
	Subunits of NADH dehydrogenase	ndhA*, ndhB*(2), *ndhC, ndhD, ndhE, ndhF, ndhG, ndhH, ndhI, ndhJ, ndhK*
	Subunits of cytochrome b/f complex	petA, petB*, petD*, petG, petL, petN
	Subunits of ATP synthase	atpA, atpB, atpE, atpF*, atpH, atpI
	Large subunit of rubisco	*rbcL*
	Subunits photochlorophyllide reductase	–
Self-replication	Proteins of large ribosomal subunit	rpl14, rpl16*, rpl2*(2), *rpl20, rpl22, rpl23*(2), *rpl32, rpl33, rpl36*
	Proteins of small ribosomal subunit	rps11, rps12**(2), rps14, rps15, rps16*, rps18, rps19, rps2, rps3, rps4, rps7(2), *rps8*
	Subunits of RNA polymerase	rpoA, rpoB, rpoC1*, rpoC2
	Ribosomal RNAs	*rrn16*(2), *rrn23*(2), *rrn4.5*(2), *rrn5*(2)
	Transfer RNAs	trnA-UGC*(2), trnC-GCA, trnD-GUC, trnE-UUC, trnF-GAA, trnG-UCC*, trnH-GUG, trnI-CAU(2), trnI-GAU*(2), trnK-UUU*, trnL-CAA(2), trnL-UAA*, trnL-UAG, trnM-CAU, trnN-GUU(2), *trnP-UGG, trnQ-UUG, trnR-ACG*(2), *trnR-UCU, trnS-GCU, trnS-GGA, trnS-UGA, trnT-GGU, trnT-UGU, trnV-GAC*(2), trnV-UAC*, trnW-CCA, trnY-GUA, trnfM-CAU
Other genes	Maturase	*matK*
	Protease	clpP**
	Envelope membrane protein	*cemA*
	Acetyl-CoA carboxylase	*accD*
	c-type cytochrome synthesis gene	*ccsA*
	Translation initiation factor	–
	other	pafI**, pafII
Genes of unknown function	Conserved hypothetical chloroplast ORF	*ycf1, ycf15*(2), *ycf2*(2)

Gene*, Gene with one intron; Gene**, Gene with two introns; Gene (2), Number of copies of the multi-copy gene.

### 3.2 Codon preference analysis

We estimated codon usage profiles for nine *Primula* species (**Figure 2**). Sixty-four codons were used in all 9 *Primula* plants, encoding 21 amino acids, and the protein-coding genes coded for codons 50, 518 (*P. secundiflora*) to 51, 551 (*P. gemmifera*). UGA, UAG, and UAA served as stop codons. Across all nine Primula species, UAA was the most preferentially used stop codon, with RSCU values ranging from 1.21 (*P. wawushanica*) to 1.30 (*P. meishanensis*). Leu (L), Arg (R), and Ser (S) had the highest number of codons, all amounting to six; Met (M) and Trp (W) each had only 1 codon, AUG and UGG, respectively, which were the least abundant. The codon with the highest number of occurrences was UUU encoding Phe (F) with 2245 (*P. purdomii*) to 2337 (*P. fangii*) codons; GCG encoding Ala (A) was the codon with the lowest number of codons for the 9 *Primula* species, with numbers ranging from 220 (*P. meishanensis*) to 263 (*P. fangii*).

**Figure 2:**
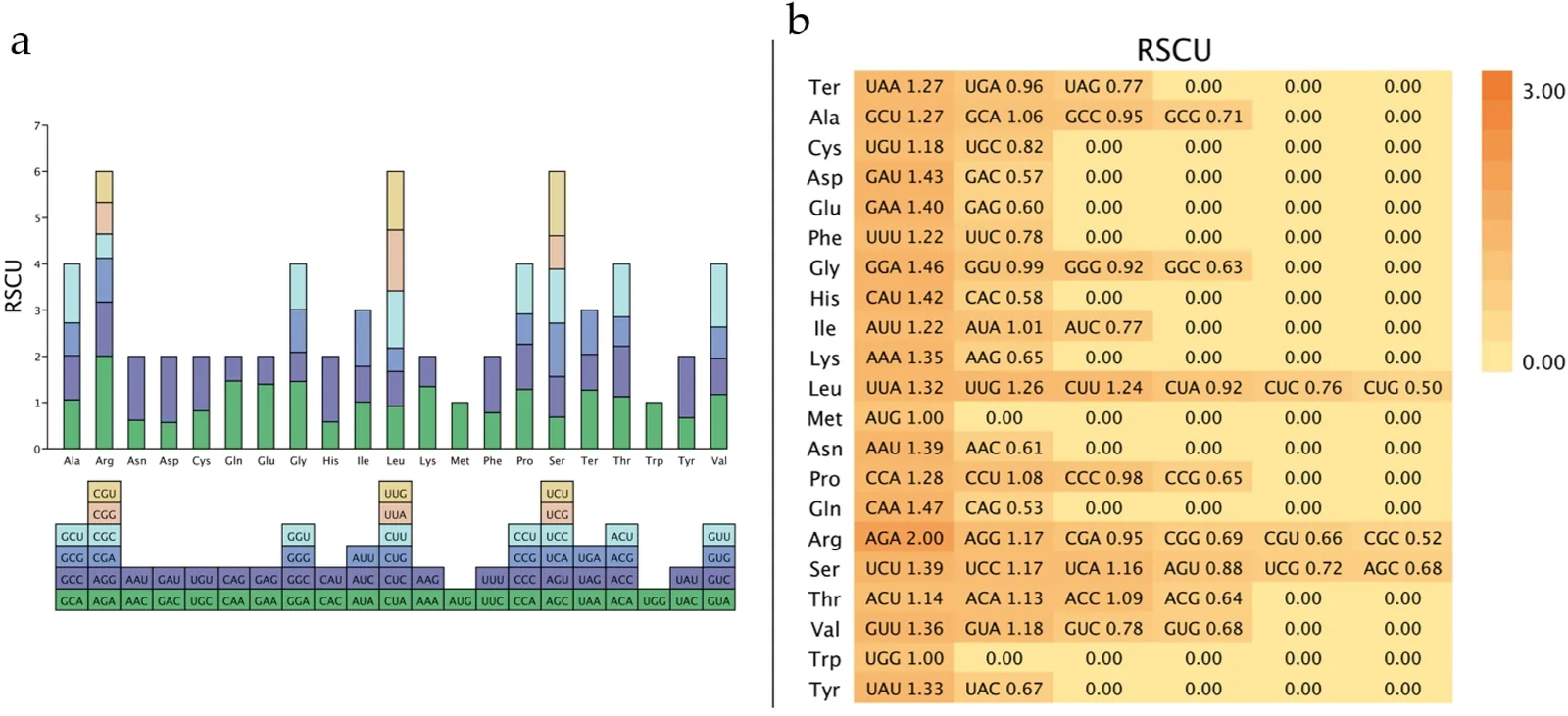
Relative synonymous codon usage (RSCU) in *Primula* plants. **(a)** Stacked bars of relative synonymous codon usage (RSCU), taking *P. fangii* as an example. **(b)** heatmap displaying RSCU values, taking *P. fangii* as an example.

The number of preferred codons (RSCU ≥ 1.00) per species ranged from 30 (*P. fangii*) to 34 (*P. purdomii*) of the 9 *Primula* species. RSCU patterns across the nine *Primula* species revealed a consistent bias toward A/U over G/C at the third codon position, which is consistent with previous findings from other angiosperm chloroplast genomes (**Supplementary Table 2**).

To further disentangle the evolutionary forces shaping codon usage bias, ENC-plots and neutrality plots were constructed for all nine *Primula* species (**Figures 3**, **4**). In ENC plots, the x-axis represented GC content at synonymous third codon positions (GC3s), the y-axis denoted the effective number of codons (ENC), and the solid curve indicated theoretical ENC values predicted under strictly neutral mutational conditions without selective constraints. Gene distribution patterns were highly congruent among all nine species: nearly all protein-coding genes clustered within a GC3s range of 0.2–0.4, confirming pervasive AT enrichment at third synonymous codon sites in *Primula* chloroplasts. Most gene data points fell distinctly below the theoretical neutral curve, demonstrating that natural selection functions as the predominant driver of codon usage bias, while compositional constraints derived from mutational pressure exert only a secondary, weak effect. A small set of outlier genes with ENC < 35 was detected in each species, indicative of intensified translational selective pressures acting on these loci; several species also harbored minor outlier genes with GC3s > 0.4, revealing unique base-composition signatures at the third codon position for a small subset of coding sequences. Only trivial interspecific disparities were observed in gene dispersion and outlier abundance, and the core evolutionary mechanisms governing codon bias remained conserved across all sampled *Primula* taxa.

**Figure 3:**
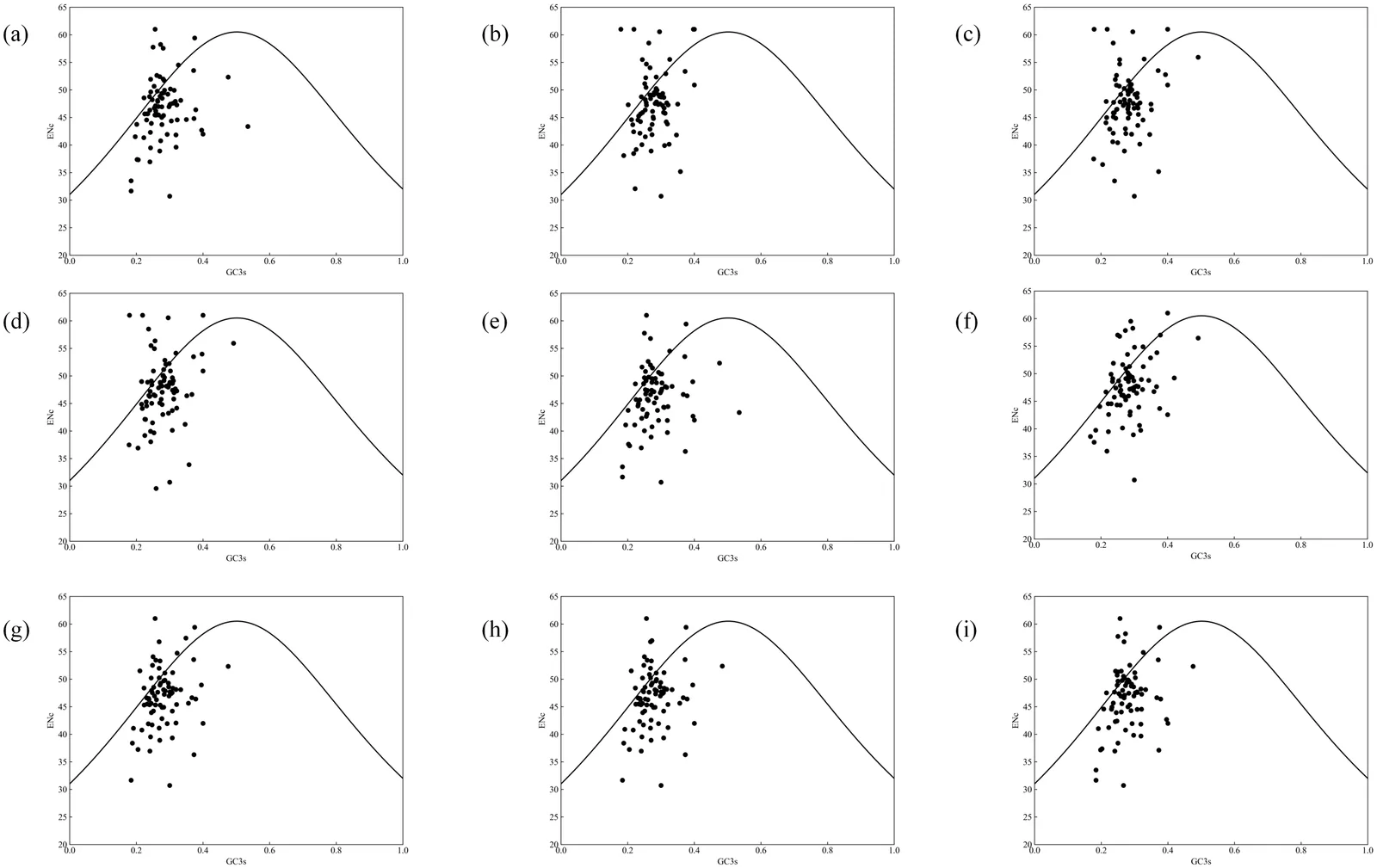
ENc-plot analysis of chloroplast genomes nine Primula species. **(a)** *P. dejuniana*; **(b)** *P. fangii*; **(c)** *P. flava*; **(d)** *P. gemmifera*; **(e)** *P. meishanensis*; **(f)** *P. polyneura*; **(g)** *P. purdomii*; **(h)** *P. secundiflora*; **(i)** *P. wawushanica*.

**Figure 4:**
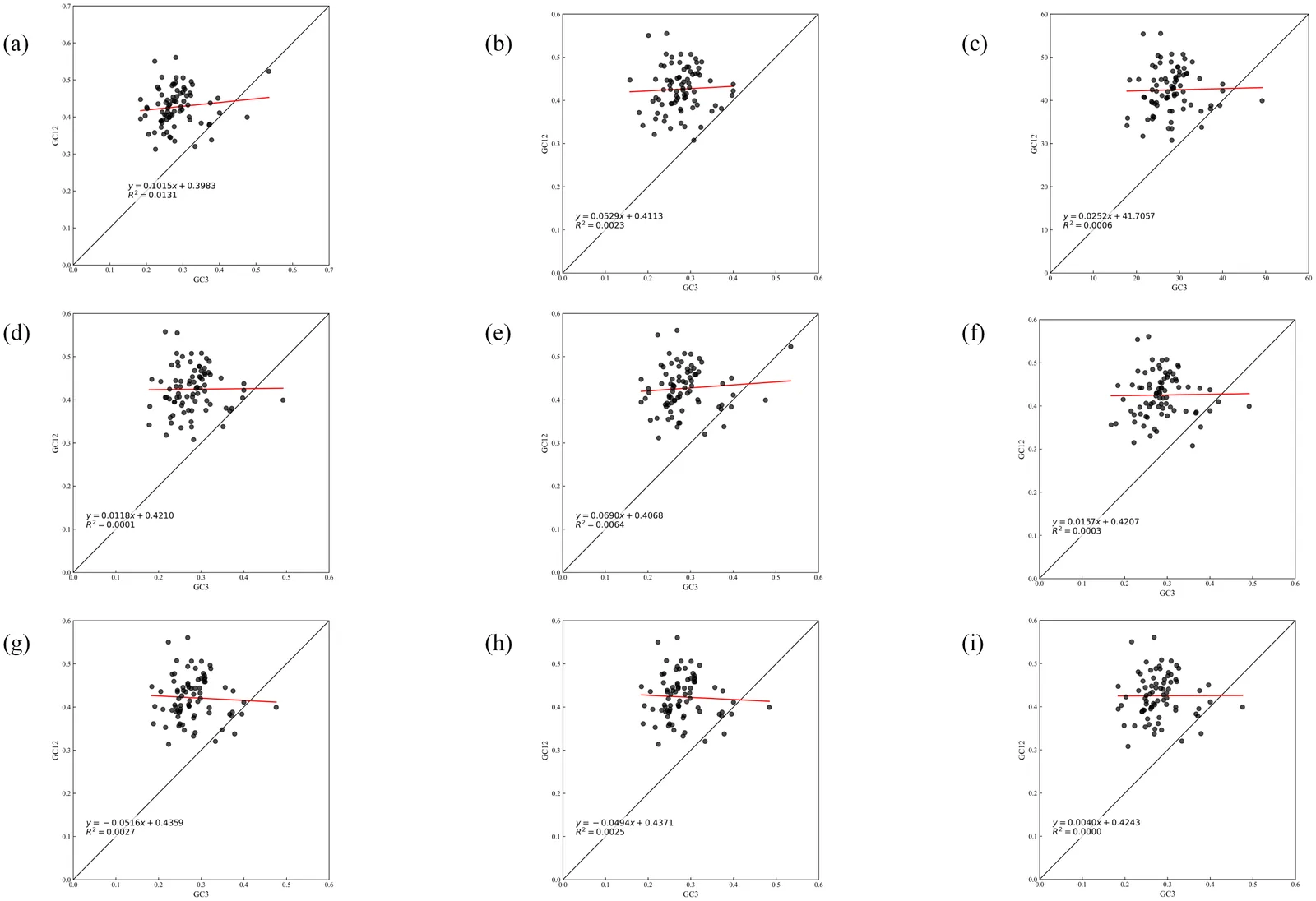
Neutrality plot analysis of chloroplast genomes nine *Primula* species. **(a)** *P. dejuniana*; **(b)** *P. fangii*; **(c)** *P. flava*; **(d)** *P. gemmifera*; **(e)** *P. meishanensis*; **(f)** *P. polyneura*; **(g)** *P. purdomii*; **(h)** *P. secundiflora*; **(i)** *P. wawushanica*.

Neutrality plots were generated with GC12 on the y-axis and GC3 on the x-axis, where the diagonal line marked the expected correlation pattern under neutral mutation. All nine species displayed nearly identical neutrality profiles: linear regression yielded extremely low coefficients of determination (R^2^ < 0.014 for all taxa), reflecting negligible linear correlation between GC12 and GC3. Fitted regression lines were nearly horizontal with slopes far less than 1. GC12 values were tightly restricted within a narrow range of 0.40–0.45 across all species, while GC3 exhibited much broader variation. This pattern demonstrates that the first and second codon positions are subject to strong purifying selection that rigidly conserves their base composition, whereas the third codon position tolerates greater mutational fluctuation. Minimal interspecific variation was observed in regression slopes and R^2^ values. Collectively, neutrality plot results corroborate the inferences drawn from ENC-plots: natural selection dominates the evolution of chloroplast codon usage bias in *Primula*, and mutational bias contributes minimally to observed compositional variation.

### 3.3 Repeat sequences and SSR analysis

In this study, SSR analysis was performed on the nine *Primula* plastomes. A total of 433 SSR loci were obtained (**Figure 5**; **Supplementary Table 3**). The SSRs comprised 323 mononucleotide repeats (1-mers; 74.6%), 50 dinucleotide repeats (2-mers; 11.5%), 10 trinucleotide repeats (3-mers; 2.3%), 40 tetranucleotide repeats (4-mers; 9.2%), 4 pentanucleotide repeats (5-mers; 0.9%), and 6 hexanucleotide repeats (6-mers; 1.4%). Among the *Primula* species, *P. purdomii*, *P. secundiflora*, and *P. wawushanica* harbored only A/T mononucleotide repeats, while the remaining six *Primula* species contained C/G motifs in addition to A/T repeats. AT/TA dinucleotide repeats were detected in all nine taxa. Trinucleotide repeats were present in six species, absent only in *P. flava*, *P. purdomii*, and *P. secundiflora*. Tetranucleotide repeats were found in all nine species, with the AATA/TATT motif being the most predominant. Pentanucleotide repeats were restricted to *P. flava*, *P. gemmifera*, *P. purdomii*, and *P. secundiflora*, and hexanucleotide repeats were identified in *P. dejuniana*, *P. fangii*, *P. flava*, and *P. gemmifera*.

**Figure 5:**
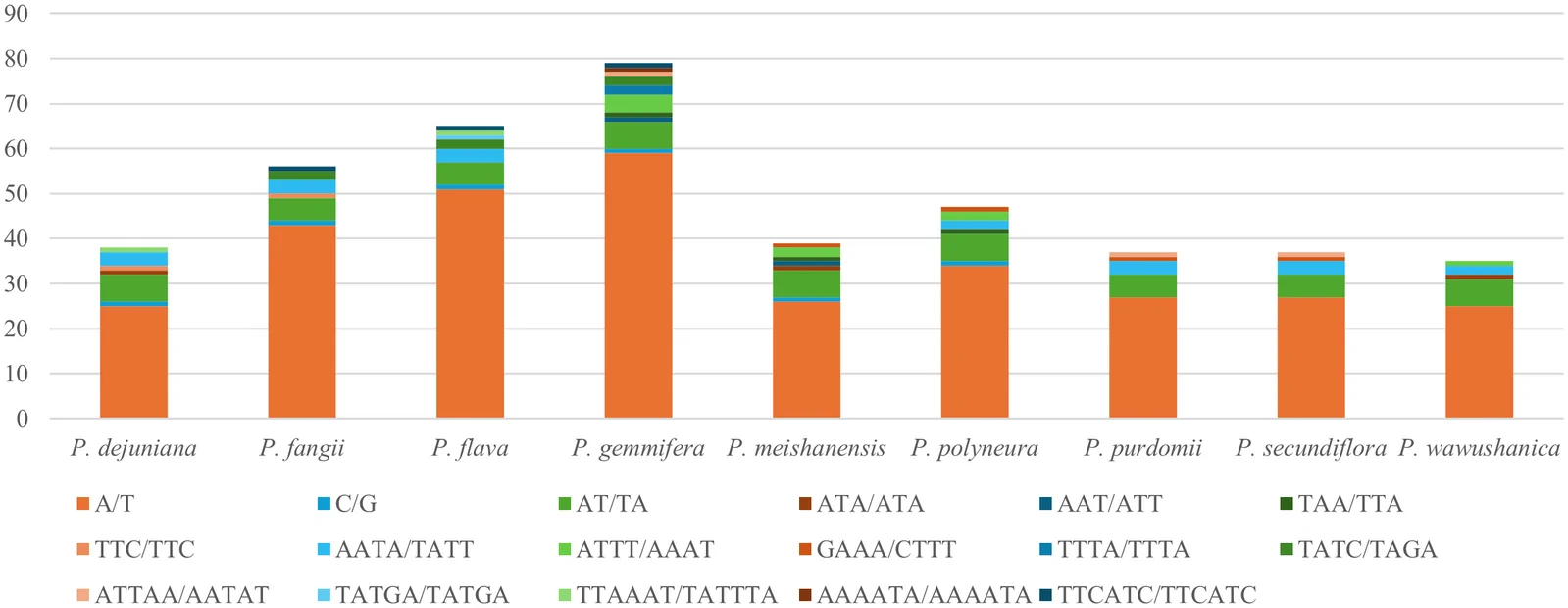
Types and numbers of simple sequence repeats in the cp genomes of nine *Primula* species.

In this study, dispersed long repeat sequences in the cp genomes of nine *Primula* species were counted (**Figure 6**; **Supplementary Table 4**). A total of 242 pairs of repetitive sequences were identified, as follows: 101 pairs (41.7%) of forward repeats (F), 138 pairs (57.0%) of palindromic repeats (P), and 3 pairs (1.2%) of reverse repeats (R). Of these, reverse repeats were found only in *P. flava* and *P. gemmifera*, with one and two pairs, respectively; forward repeats and palindromic repeats were found in all nine *Primula* species. The fewest repetitive sequences were found in *P. wawushanica* with 22 pairs, including 13 pairs of forward repeats and 9 pairs of palindromic repeats. The highest number of repetitive sequences was found in *P. gemmifera* with a total of 34 pairs, including 12 pairs of forward repeats, 20 pairs of palindromic repeats, and 2 pairs of reverse repeats. The least number of forward repeats were in *P. meishanensis*, with 9 pairs; the most were in *P. purdomii*, *P. secundiflora*, and *P. wawushanica*, all with 13 pairs.

**Figure 6:**
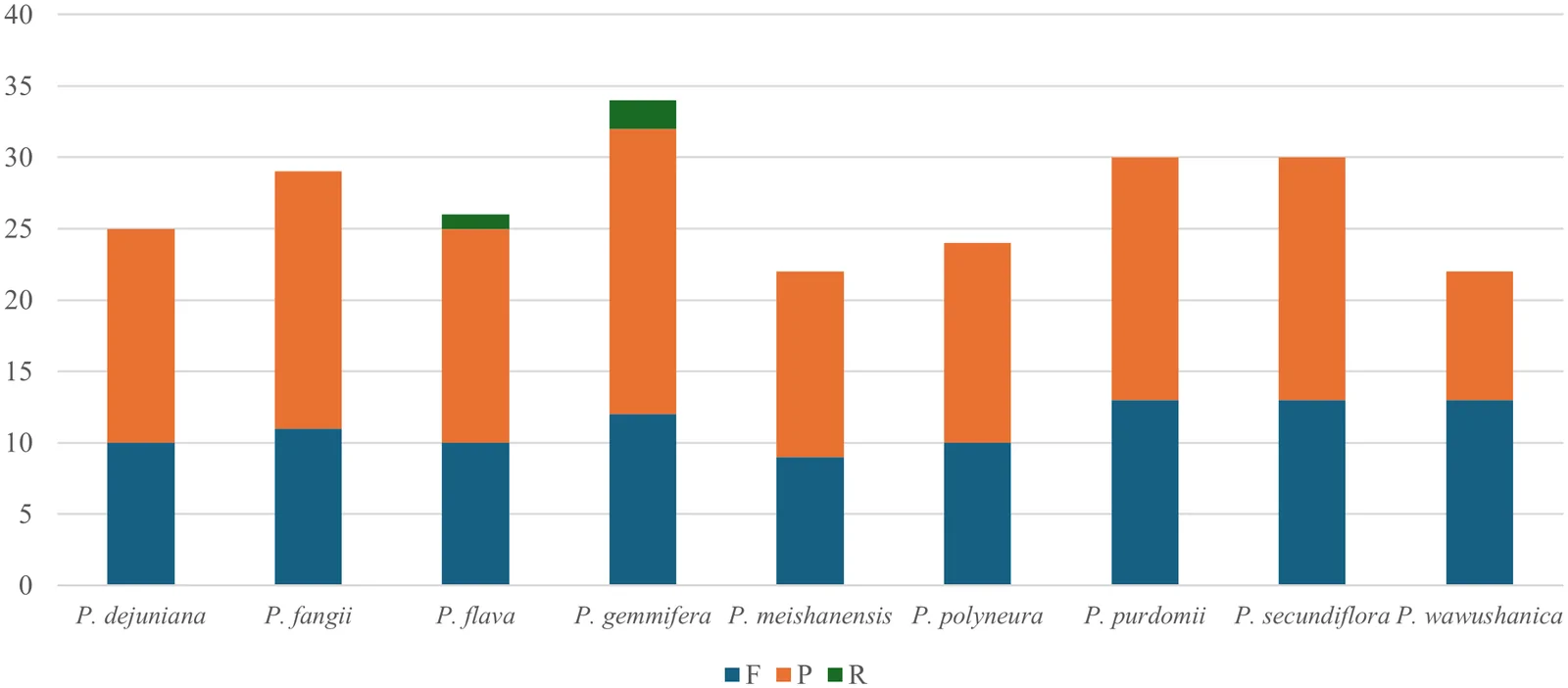
Types and numbers of large repeat sequences in the cp genomes of nine *Primula* species.

### 3.4 IR expansion and contraction

We systematically compared the boundary positions of the large single-copy (LSC), small single-copy (SSC), and inverted repeat (IRa/IRb) regions across the chloroplast genomes of nine *Primula* species (**Figure 7**; **Table 3**). All nine genomes exhibited a typical quadripartite structure, with high conservation in the total gene count and sequence length of LSC, SSC and IR regions across taxa. Nonetheless, extensive lineage-specific variations were detected at the LSC/IRs/SSC regions, primarily manifested in variable IR length and shifted positions of boundary flanking genes. The length of a single IR copy ranged from 25, 175 bp (*P. dejuniana*) to 26, 147 bp (*P. wawushanica*). Several genes exhibited conserved junction locations across all species: *ndhF* consistently crossed the IRb/SSC boundary, while *ycf1* was universally distributed at the SSC/IRa junction with variable extension lengths into IRa; the *trnH* gene adjacent to the IRa/LSC border was separated from the IRa terminus by only 3–14 bp.

**Figure 7:**
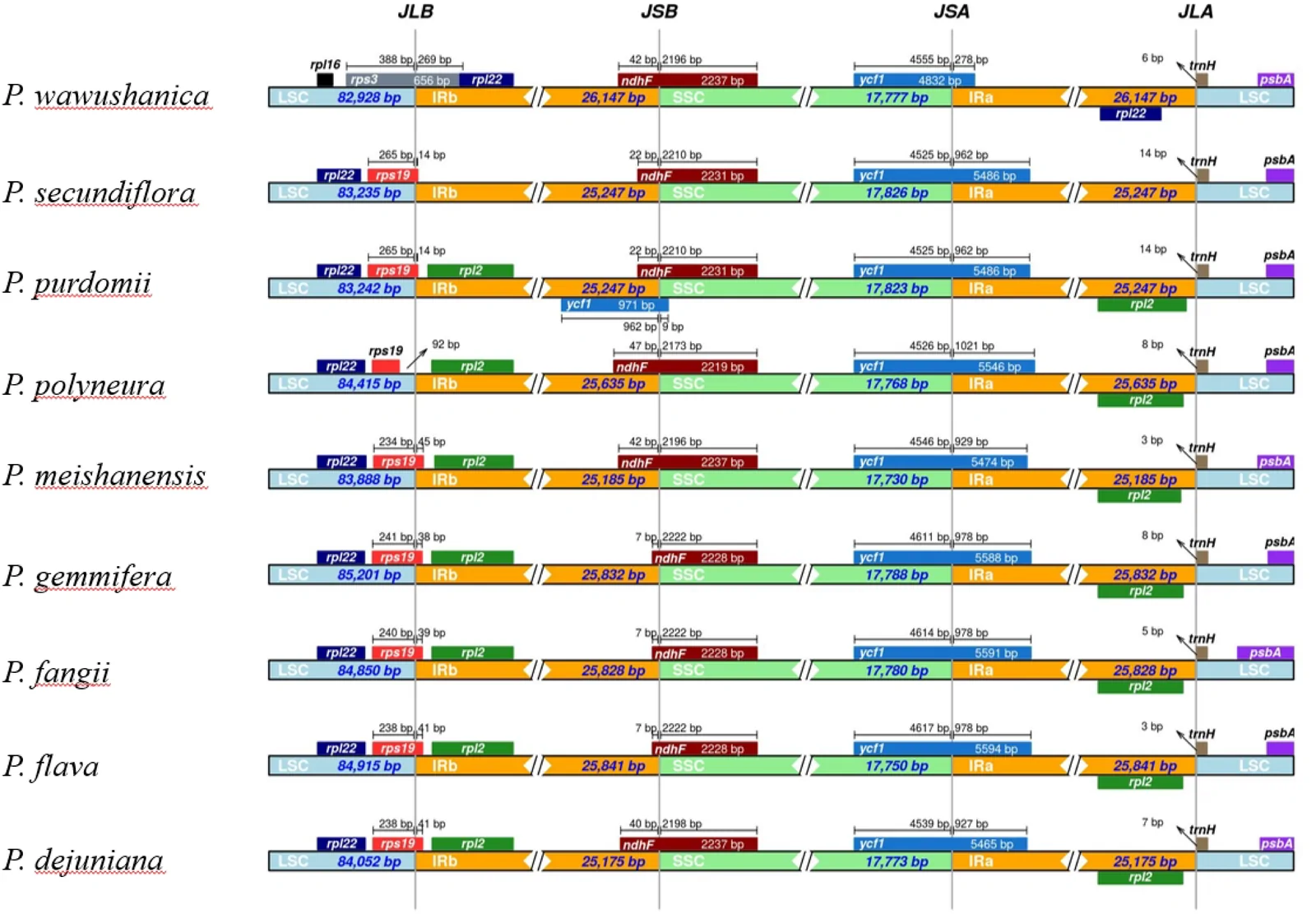
Comparisons of the borders of LSC, SSC, and IR regions among nine *Primula* chloroplast genomes.

**Table 3:** Distribution of core genes at IR boundaries of cp genomes from nine *Primula* species.

Species	LSC/IRb junction	IRb/SSC junction	SSC/IRa junction	IRa/LSC junction
*P. wawushanica*	*rpl16, rps3, rpl22*	*ndhF*	*ycf1*	*trnH, psbA, rpl22*
*P. secundiflora*	*rpl22, rps19*	*ndhF*	*ycf1*	*trnH, psbA*
*P. purdomii*	*rpl22, rps19, rpl2*	*ndhF, ycf1*	*ycf1*	*trnH, psbA, rpl2*
*P. polyneura*	*rpl22, rps19, rpl2*	*ndhF*	*ycf1*	*trnH, psbA, rpl2*
*P. meishanensis*	*rpl22, rps19, rpl2*	*ndhF*	*ycf1*	*trnH, psbA, rpl2*
*P. gemmifera*	*rpl22, rps19, rpl2*	*ndhF*	*ycf1*	*trnH, psbA, rpl2*
*P. fangii*	*rpl22, rps19, rpl2*	*ndhF*	*ycf1*	*trnH, psbA, rpl2*
*P. flava*	*rpl22, rps19, rpl2*	*ndhF*	*ycf1*	*trnH, psbA, rpl2*
*P. dejuniana*	*rpl22, rps19, rpl2*	*ndhF*	*ycf1*	*trnH, psbA, rpl2*

Multiple lineage-specific structural variations, including gene loss, duplication and boundary shift, were detected among species. Two taxa (*P. wawushanica*, *P. secundiflora*) lacked the *rpl2* gene, whereas only *P. wawushanica* carried duplicated *rpl22* in both *IRa* and *IRb* and lost the *rps19* gene. Unique to *P. purdomii*, an extra copy of *ycf1* was identified at the IRb/SSC junction. The *rps19* of *P. polyneura* shifted 92 bp inward into the LSC region rather than anchoring at the LSC/IRb border. In addition, *P. wawushanica* displayed a distinctive gene arrangement at the LSC/IRb junction that was absent in other congeners. Detailed information on boundary gene composition, IR length and intergenic intervals for each species is summarized in **Supplementary Table 3** for straightforward interspecific comparison. The *rpl22-rps19* genes were located at the boundary of the LSC/IRb region, and *psbA* and *trnH* genes were consistently located in the LSC region in terms of the structure of the cp genome and the *trnH* genes were associated with the LSC/IRa boundary; the *rps19* genes were located in the LSC/IRa region. IRA boundary is very close, only 3 (*P. meishanensis*)-14 (*P. secundiflora and P. purdomii*) bp are present; *ndhF* genes are always present at the IRB/SSC boundary; and, except for *P. wawushanica*, which has two copies of the rpl22 gene located in the IRA and the IRB, the other eight species of *Primula rpl22* gene were all located in the LSC region; the *ycf1* gene was always located in the SSC/IRA boundary region, the proportion of which varied from species to species, but in *P. purdomii*, two copies of the *ycf1* gene were present at the IRB/SSC boundary as well; and, except for the presence of a deletion of the *rpl2* gene in *P. wawushanica* and *P. secundiflora*, the remaining seven species of *Primula rpl2* gene all have two copies, located in IRA and IRB; notably, the rps19 gene of *P. polyneura* is located in the LSC region, separated from the LSC/IRB boundary by 92 bp, and the *rps19* genes of the remaining seven *Primula* species, except for *P. wawushanica*, which has *rps19* gene deletion, are all located in the LSC/IRB boundary region; furthermore, the *rpl16* gene is located in the LSC region and *rps3* is located in the LSC/IRB boundary is found only in *P. wawushanica*.

### 3.5 Analysis of chloroplast genome sequence divergence

This study utilized mVISTA to compare the cp genome sequences of nine *Primula* species, using *Ardisia polysticta* as a reference. The results indicated that the cp genome sequences of the nine *Primula* species are highly similar (**Figure 8**). The non-coding regions exhibit greater variation than the coding regions, and the LSC and SSC regions show more differences compared to the IR regions. A certain degree of high differentiation was observed in regions such as *matK*, *rpoC2*, *accD*, *petB*, *petD*, *infA*, *rpl16*, *rps19*, *trnI-GAU*, *ndhF*, *ccsA*, and *ndhA*. Compared to *Ardisia polysticta*, the infA gene varies the most in the LSC region, ycf1 shows the most significant variation in the IR region, and ndhA exhibits the most notable variation in the SSC region.

**Figure 8:**
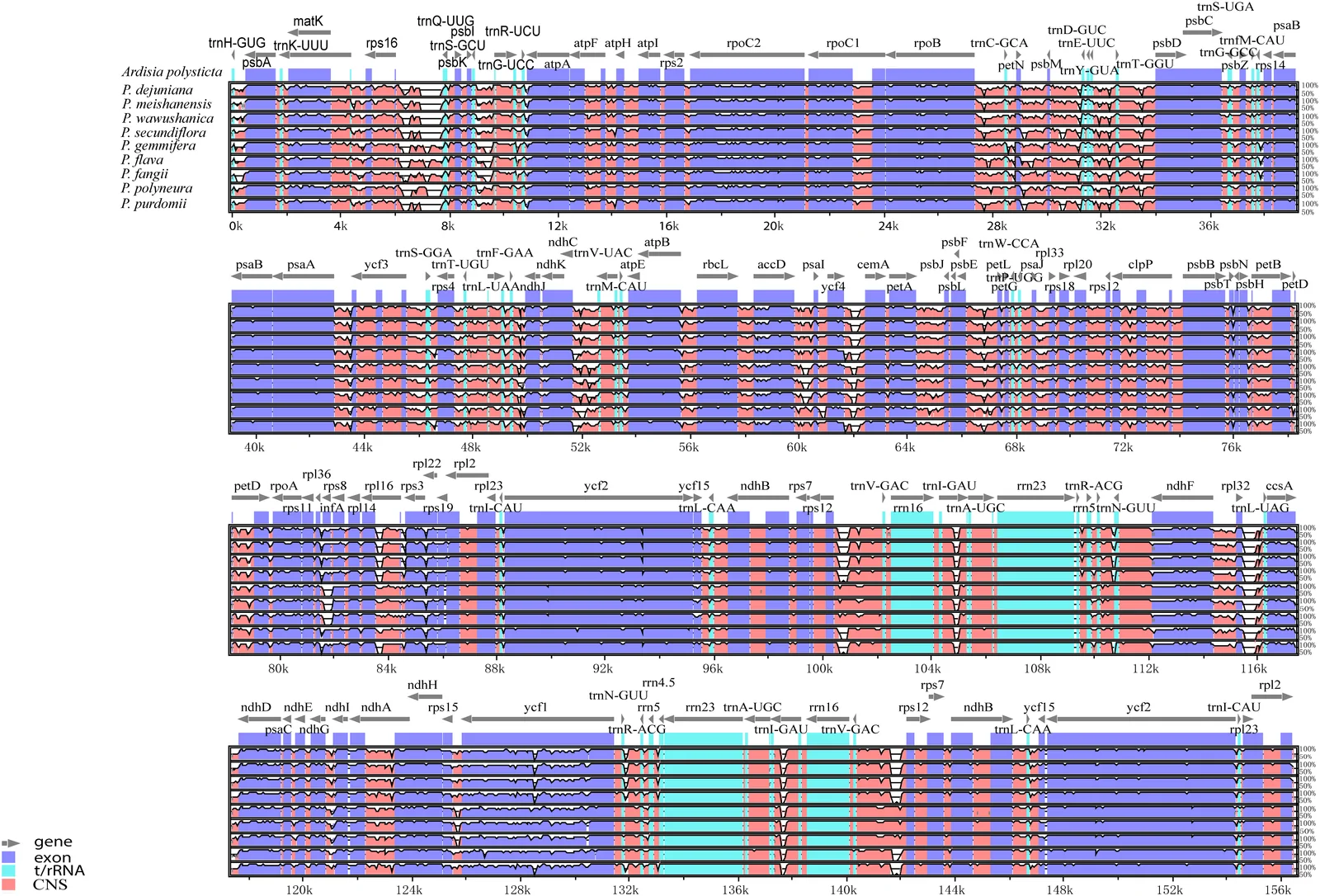
Comparative plots based on the sequences identity of cp genome of nine *Primula* species, using *Ardisia polysticta* as the reference genome.

### 3.6 Basic characteristics of highly variable area segments

Nucleotide polymorphism analysis was performed on the nine *Primula* species in this study. The results showed markedly higher nucleotide diversity in the LSC and SSC regions relative to the more conserved IR regions. The Pi values across the entire *Primula* cp genome ranged from 0 to 0.09319. Six highly variable regions (Pi > 0.05785) were detected, named as follows: *rps16_1-trnQ*, *psbM-trnD*, *rpl32-trnL*, *rpl32*, *ndhF-rpl32*, and *ycf1*. Among these, the first two are located in the LSC region, and the last four are located in the SSC region (**Figure 9**). These six hypervariable regions represent promising candidate molecular markers for the identification of *Primula* species. Further primer design and experimental validation will be conducted in future work.

**Figure 9:**
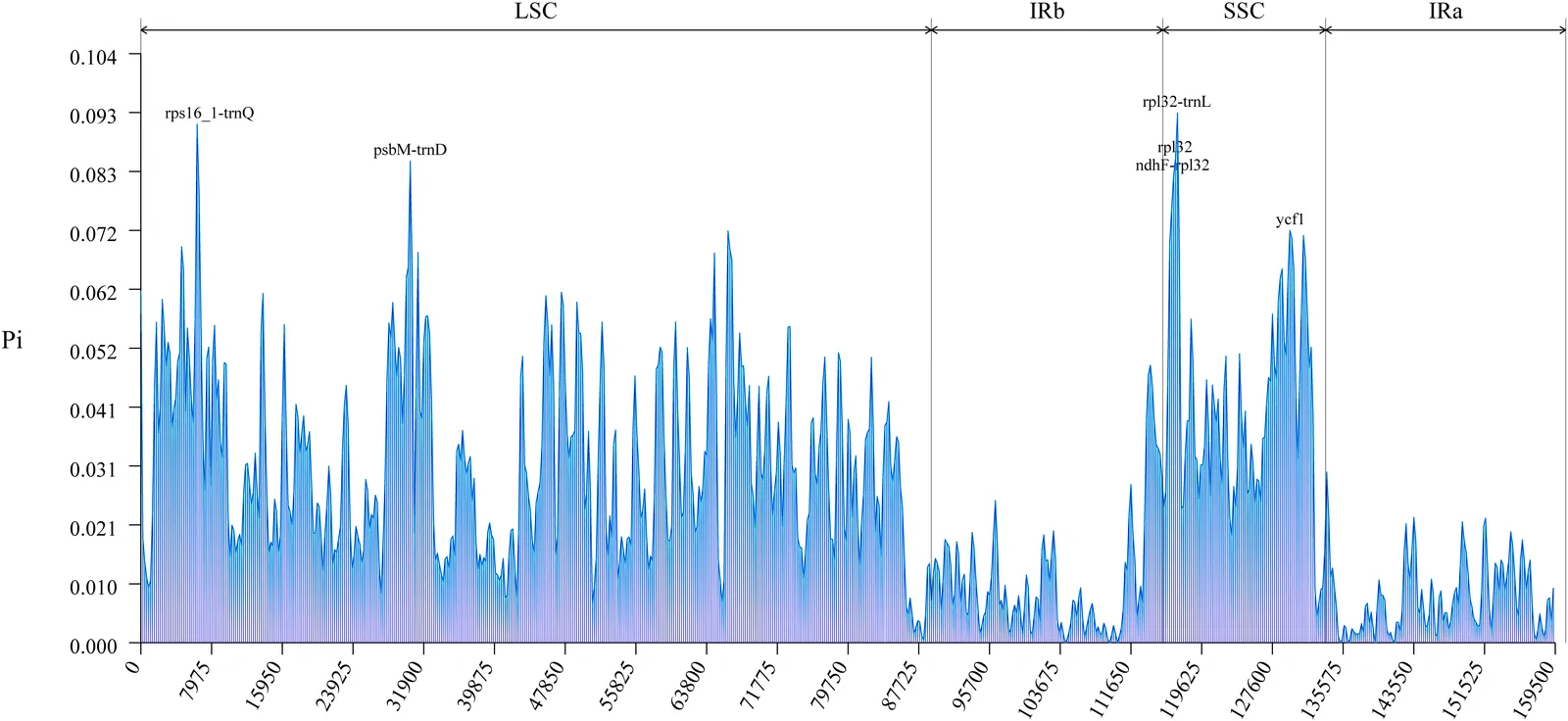
Nucleotide diversity (Pi) analysis of cp genomes of 9 *Primula* species.

### 3.7 Phylogenomic analysis and divergence time estimation

The phylogenomic tree of *Primula* was reconstructed using the identical concatenated plastid protein-coding CDS alignment matrix, with three outgroups (*Ardisia polysticta*, *Androsace bulleyana*, *Androsace erecta*) designated to root the phylogenetic framework. Consistent and well-supported topological relationships were recovered across the whole tree, with most internal nodes bearing high posterior probability and bootstrap support values shown beside each branch (**Figure 10**; **Supplementary Figures S1**, **S2**). The phylogenetic tree showed that all samples exhibited 21 of the 24 groups of the genus *Primula*, which can be divided into three main branches. Branch A diverged earliest within core *Primula*, comprising species from Sects. *Auganthus*, *Obconicolisteri*, *Bullatae*, *Carolinella*, *Malvacea*, *Aleuritia*, *Cortusoides* and *Monocarpicae*. The target species *Primula polyneura* (marked with a black star) was embedded within Sect. *Aleuritia* in this early-diverged lineage; branch B consists of nine sections (Sects. *Primtula*, *Minutissimae*, *Souliei*, *Sikkimensis*, *Aleuritia*, *Denticulata*, *Capitatae*, *Muscarioides*, *Soldanelloides*). Three star-marked species were nested here: *P. fangii*, *P. flava* and *P.gemmifera*, all belonging to Sect. *Aleuritia*, revealing multiple independent radiations of this section across separate subclades. And branch C represented the youngest and largest subclade, encompassing Sects. *Crystallophlomis*, *Ranunculoides*, *Proliferae*, *Sikkimensis*, *Amethyatina*, *Petiolares*. This clade harboured five remaining target species: *P. meishanensis*, *P. dejuniana* and *P. wawushanica* are closely related in Sect. *petiolares*; *P. secundiflora* and *P. purdomii* in Sect. *Proliferae.*This study estimated the divergence times of 57 species of *Primula*. The stem divergence between outgroup genera (*Androsace*) and the crown group of *Primula* was dated to approximately 27.21 Mya (95% HPD: 18.72–35.82 Mya) during the late Oligocene. The split of Clade A from the ancestor of Clade B + Clade C occurred at 19.59 Mya (95% HPD: 13.19–25.89 Mya, Early Miocene). The focal species *P. polyneura* in Clade A originated at 4.86 Mya (2.99–6.85 Mya, late Miocene–Pliocene). The separation between Clade B and Clade C was estimated at 17.62 Mya (95% HPD: 11.82–22.66 Mya, Early Miocene). The three star-marked species within Clade B diversified between 1.10–5.84 Mya, concentrated in the late Miocene to Pleistocene. Clade C began its primary radiation at 8.94 Mya (95% HPD: 5.53–12.83 Mya, late Miocene). The sectional lineages within Clade C split sequentially from the late Miocene to Pleistocene. The focal species of Sect. *Proliferae* (*P. poissonii*, *P. secundiflora*) diverged at ~3.47 Mya, while the Sect. *Petiolares* target species diversified during 1.55–2.12 Mya, corresponding to the late Pliocene and early Pleistocene.*P. meishanensis*, *P. dejuniana*, and *P. wawushanica* diverged between 0.86 and 2.83 Mya; and the most recent divergence was detected between *P. secundiflora* and *P. purdomii* at 0.11 Mya [95% HPD: 0.03–0.23 Mya]. Our age estimate carries relatively broad uncertainty intervals, and complementary evidence from nuclear genomic datasets would help corroborate these dating results. Divergence time analyses built solely on plastid sequences suggest that the genus *Primula* initiated its rapid radiation at roughly 19.59 Mya (95% HPD: 13.19–25.89 Mya, Early Miocene). The chronological framework recovered in this work lays a fundamental dataset to facilitate future investigations into the evolutionary history of *Primula*.

**Figure 10:**
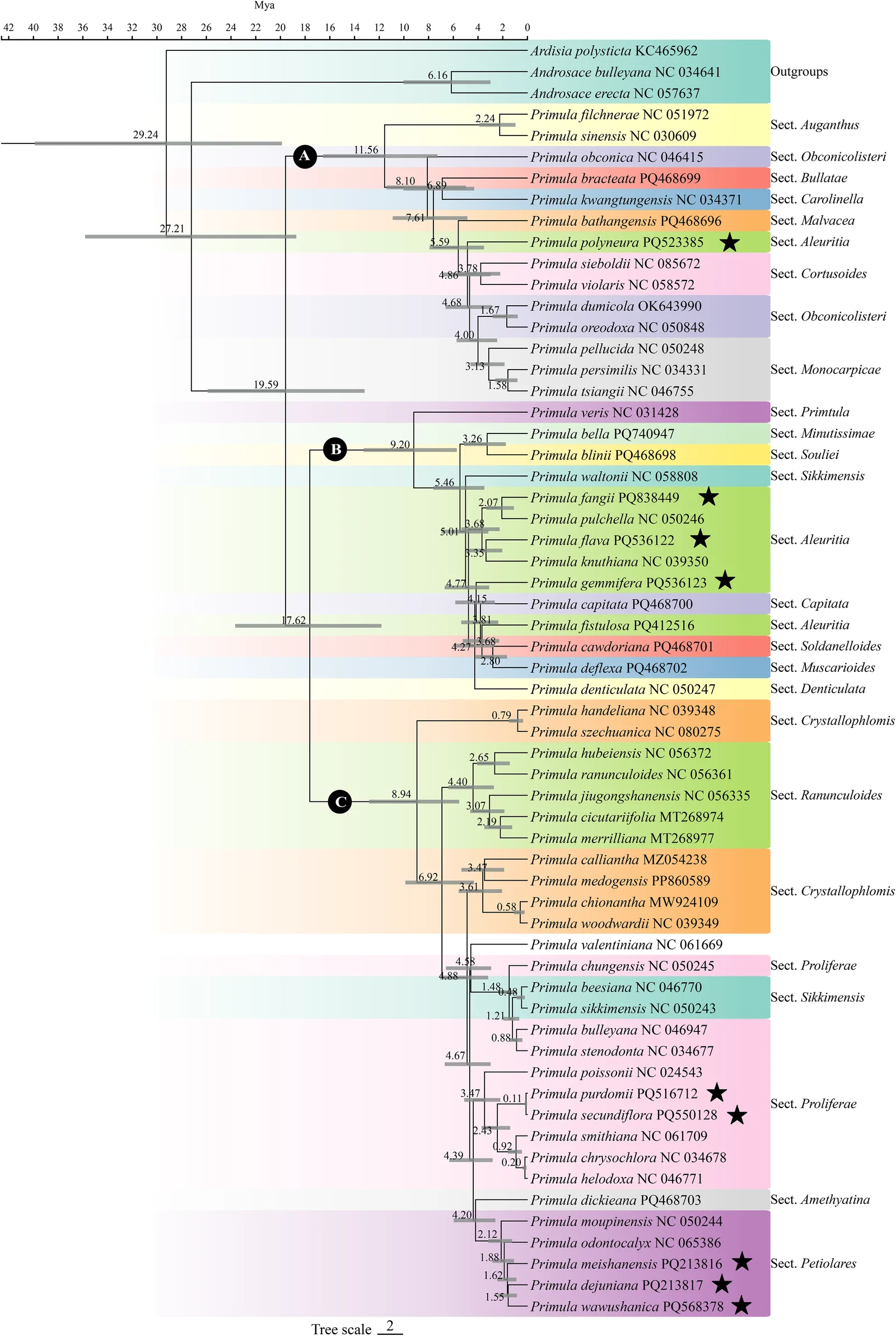
Phylogeny and time scale of *Primula* species based on concatenated plastid CDS sequences. **(A–C)** denote the three major clades within core *Primula*. Species in black stars indicate the nine *Primula* species studied here. The grey striped area represents the posterior probability density of the 95% highest posterior density (HPD) interval.

### 3.8 Ancestral character state reconstructions

Ancestral character reconstruction was performed for four core morphological traits of *Primula*, including inflorescence type, style morph, corolla color, and calyx morphology. Ancestral state estimations displayed in the main figures were generated under the ARD evolutionary model (**Figure 11**), while complementary reconstructions based on the ER and SYM models were presented as Supporting **Supplementary Figures S4**, **S5** for comparative reference. Overall, highly congruent ancestral state patterns were recovered across the three models. The results suggest that the umbel represents the likely ancestral inflorescence type of *Primula*, indicating that the compact umbellate floral structure had already formed during the early diversification of the genus. Subsequently, capitulum and raceme inflorescences evolved independently in several lineages; the reconstructions consistently show that heterostyly is the ancestral condition in *Primula*, traceable to the most recent common ancestor of the genus. By contrast, homostyly has originated repeatedly in multiple clades; the purple corolla is inferred as the ancestral color, whereas yellow and white corollas evolved independently multiple times during later diversification. For calyx morphology, campanulate calyx is inferred to be the ancestral state of the genus, and narrowly campanulate calyx represents a derived form that emerged later in the evolutionary process.

**Figure 11:**
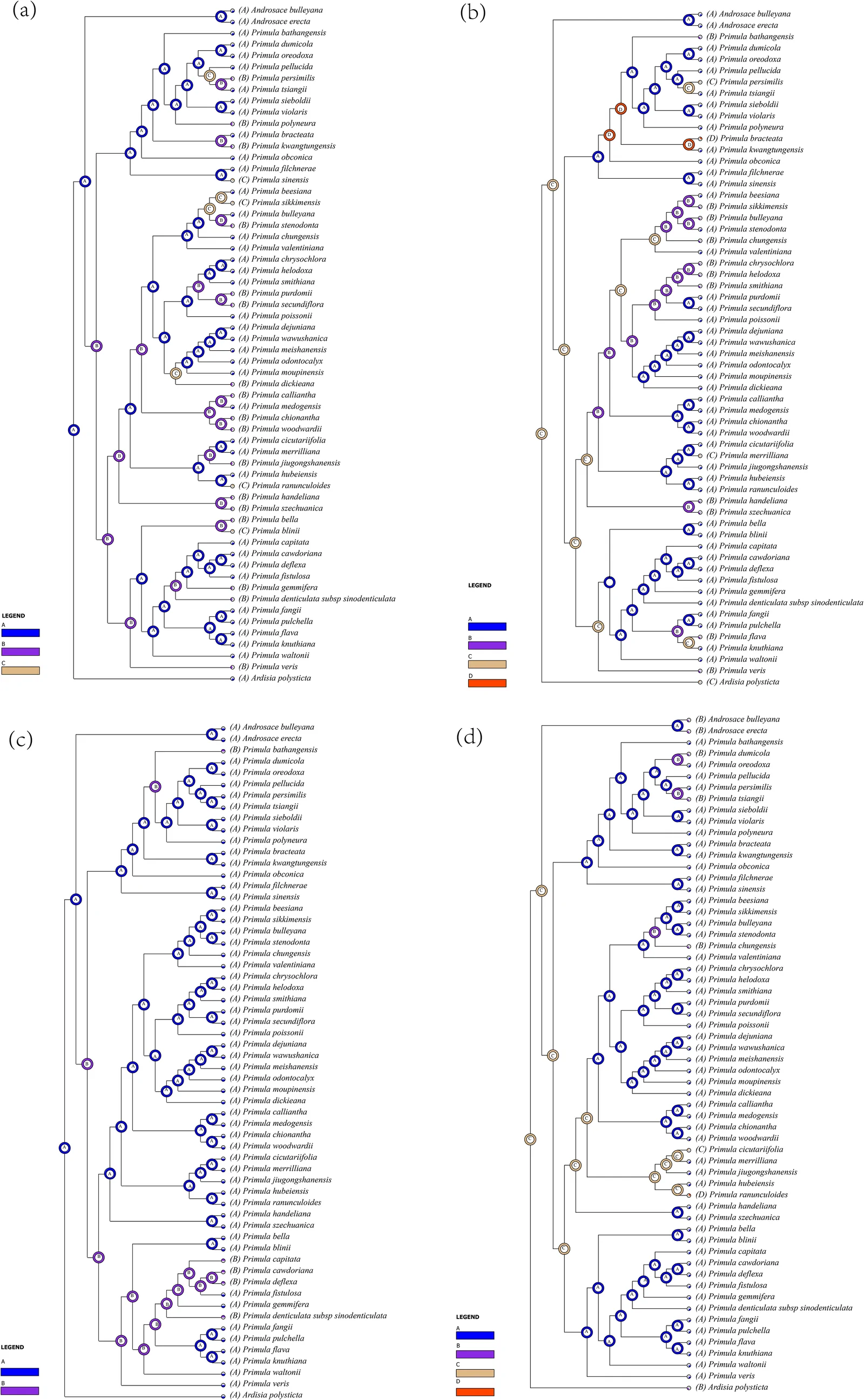
Ancestral state reconstruction of four morphological traits of *Primula* based on the all-rates-different (ARD) Mk model. **(a)** calyx; **(b)** corolla color; **(c)** inflorescence; **(d)** style.

## 4 Discussion

### 4.1 Evolution of the chloroplast genomes

Nine *Primula* cp genomes were assembled and uniformly exhibited the canonical angiosperm quadripartite architecture, usually consisting of an LSC, SSC, and two IR regions (Daniell et al., 2016; Robbins and Kelly, 2023). Length varied narrowly from 151, 555 to 154, 653 bp, with GC-content between 36.85 and 37.08%, corroborating the high structural conservation reported for the genus (Li, 2022). While plastid genomes are generally conserved across angiosperms, evolutionary events such as gene loss and rearrangements are not uncommon (Braukmann et al., 2013; Liu et al., 2023). It was found that the infA gene was completely lost in the nine *Primula* plants studied. Plastid gene content is not uniformly conserved; we identified two notable gene losses in the *Primula* plastomes. The infA gene, essential for translation initiation complex assembly (Wicke et al., 2011), was absent from all nine species—a recurring theme in angiosperm plastome evolution (Millen et al., 2001; Liu et al., 2023; Scobeyeva et al., 2021). More taxonomically restricted was the loss of accD, encoding a key enzyme in fatty acid biosynthesis (Kode et al., 2005), which we observed in *P. dejuniana*, *P. meishanensis*, and *P. wawushanica* (**Supplementary Table 6**). This mirrors parallel losses in other monocot families (Harris et al., 2013; Jansen et al., 2007), suggesting lineage-specific relaxation of selective constraints.

Simple sequence repeats (SSRs), consisting of tandemly repeated motifs of 1–6 bp, are widely employed as molecular markers due to their abundance, polymorphism, and technical simplicity (Doorduin et al., 2011; He et al., 2012). They are widely applied in genetic mapping, gene localization, assessment of genetic diversity, molecular-assisted breeding, and germplasm identification (Zalapa et al., 2012; Ramzan et al., 2020). In this study, 433 SSRs were identified across the nine *Primula* species, most of which were mononucleotides were mononucleotide A/T SSRs, supporting the results of other studies (Kuang et al., 2011; Martin et al., 2013). Most of the detected SSRs were distributed in the LSC region of the chloroplast genome, and a few were also found in the IR region. Similar phenomena were found in other *Primula* plants and angiosperms (Luo et al., 2021; Shen et al., 2020; Xie et al., 2023b). Repeat sequences, especially those longer than 20 bp and 60 bp, are known to play important roles in chloroplast genome variation and rearrangement (Guisinger et al., 2011; He et al., 2012; Powell et al., 1995). A total of 242 long repetitive sequences, ranging from 30 to 55 bp in length, were identified in this study. The differences in the number, type and length of the repeat sequences of the nine *Primula* species reported in this paper can provide a theoretical basis for subsequent studies of molecular genetic markers in plants.

Although the IR regions are generally conserved, their boundaries may shift during evolution, leading to expansions or contractions that contribute to variation in genome size (Ren et al., 2021; Sun et al., 2017). Comparing the chloroplast genomes of the nine *Primula* species, the number and order of genes at the IR boundary were roughly similar, but there were differences. Among them, the IRa regions of all nine *Primula* species extended into the *ycf1* gene. However, the IRb region boundaries varied considerably, with the IRb boundary of *P. wawushanica* extending into the rps3 gene, and the IRb boundary of *P. polyneura* having no genes present. Compared to the other eight species, *P. wawushanica* has the longest IR region (26, 147bp), mainly because there are two copies of the *rpl22* gene. Similar IR boundary variations and copy-number differences have been documented in other *Primula* species.

According to the results of sequence divergence analysis done by mVISTA, the variation in LSC and SSC regions is significantly higher than IR regions, and the non-coding regions are more variable than coding regions. Xie et al. have similarly utilized *P. secundiflora* in their investigation of *Primula*. However, our study expands upon their work by including a broader range of *Primula* species, resulting in a more comprehensive analysis and set of results (Xie et al., 2023b). In this study, six highly differentiated gene fragments, including four intergenic spacers (*rps16_1-trnQ*, *psbM-trnD*, *rpl32-trnL*, *ndhF-rpl32*) and two coding regions (*rpl32*, *ycf1*), were preliminarily screened by analyzing polymorphic regions from complete chloroplast genomes of nine *Primula* species. The coding regions *ycf1* and *rpl32* have been frequently reported as variable molecular loci in plastid genome investigations of various plant groups (Li et al., 2023a; Liu et al., 2023; Wonok et al., 2023).

Synonymous codon usage bias universally exists in prokaryotes and eukaryotes and reflects genomic adaptive evolution (Zhang et al., 2007). It is mainly shaped by mutational pressure, natural selection and genetic drift, and plays a vital role in plant evolution (Lyu et al., 2024; Parvathy et al., 2022). In this study, codon usage analysis revealed conserved molecular evolutionary features of *Primula* chloroplast genomes. Consistent with most angiosperm plastomes, the nine *Primula* species showed obvious A/U preference at the third codon position (Li et al., 2025; Yang et al., 2023). ENC-plot and neutrality plot analyses demonstrated that natural selection predominated over mutational pressure in regulating codon usage bias. The first and second codon positions were strictly constrained by purifying selection to ensure protein functional stability, whereas the third position allowed greater nucleotide variation. The consistent codon usage patterns and evolutionary drivers across all species further confirm the high conservation and stable selective constraints of *Primula* chloroplast genomes (Li et al., 2025).

### 4.2 Phylogenetic analysis and divergence time of Chinese *Primula*

The complete chloroplast genome has become a widely utilized resource for resolving plant phylogenetic relationships and informing taxonomic classifications (Huang et al., 2022; Niu et al., 2023; Yoon et al., 2023). In this study, three phylogenetic trees were built via separate analytical pipelines from the same plastid CDS dataset: an ML tree, a BI tree and a time-calibrated MCC chronogram. Both ML and BI topologies yielded well-supported, congruent phylogenetic frameworks. The resulting phylogenetic tree revealed that all samples corresponded to 21 of the 24 recognized groups within the genus *Primula*. Due to incomplete sampling, Sect. *Cordifoliae*, Sect. *Dryadifolia* and Sect. *Pycnoloba* were not included in this analysis. Incomplete sectional sampling may affect the rooting stability of the phylogenetic tree and the estimation accuracy of divergence times at deep nodes. Therefore, the divergence time estimates and infrageneric evolutionary relationships inferred in this study are preliminary conclusions. More comprehensive sampling covering all recognized sections is required in future studies to further validate the evolutionary timeline and phylogenetic framework of *Primula*. Our phylogenetic tree showed multiple non-monophyletic sections of *Primula*, such as Sect. *Proliferae*, Sect. *Sikkimensis*, Sect. *Obconicolisteri*, Sect. *Monocarpicae*, Sect. *Cortusoides*, Sect. *Carolinella*, Sect. *Aleuritia*, Sect. *Denticulata*, and Sect. *Crystallophlomis*, which was partly or entirely confirmed by the previous viewpoints (Yan et al., 2015; Xu et al., 2020). However, the monophyly of Sects. *Proliferae*, *Sikkimensis* proposed by Yan et al. (2015) in their previous study is not supported here. Such a situation is consistent with the findings of Xie et al (Xie et al., 2023b). Non-monophyletic species are prevalent across various taxa within Primulaceae (Schmidt-Lebuhn et al., 2012; Mast et al., 2004). This study shows that *Primula* exhibits reticulate topological signatures, with multiple traditionally defined sections subdivided into geographically isolated, independent subclades scattered across different lineages. For instance, Sect. *Aleuritia* is divided into two isolated subclades located in Clade A and Clade B, separated by distinct other sections. Sect. *Sikkimensis* also exhibits a disjunct distribution, with *P. waltonii* nested in Clade B and *P. sikkimensis* placed distantly in Clade C. Similarly, Sect. *Proliferae* and Sect. *Crystallophlomis* are both fragmented into multiple disconnected lineages interspersed with unrelated sections. Such scattered, broken sectional topology suggests repeated historical reticulate evolutionary events within the genus, consistent with previous phylogenomic reports on *Primula* (Li, 2022). Even nuclear-genome-based phylogenies display blurred phylogenetic positions for densely sampled species such as *P. poissonii*, collectively indicating repeated reticulate evolutionary events throughout the genus’s evolutionary history (Liu, 2018). The phylogenetic relationships within *Primula* may be complicated by adaptive radiation driven by heterostyly, polyploidization, and natural hybridization or introgression (Guggisberg et al., 2009; Xie et al., 2017; de Vos et al., 2014). The genus is mainly pollinated by bees and butterflies; diverse floral visitors greatly raise the probability of interspecific cross-pollination (Changjiang et al., 2017). As the core diversity center of *Primula*, the Hengduan Mountains region possesses complex topography and heterogeneous microhabitats, which to a certain extent served as glacial refugia, as well as corridors and geographical barriers for species dispersal during glacial cycles (Rahbek et al., 2019; Jiaxin, 2020). Repeated upward and downward shifts of montane vegetation belts forced formerly allopatric taxa into contact (Antonelli et al., 2018; Comes and Kadereit, 1998). Combined with weak interspecific reproductive barriers within *Primula*, numerous previous studies have documented multiple natural hybridization events across this genus (Cianchi et al., 2015; Ma et al., 2014; Kato et al., 2008). Recurrent introgressive hybridization may facilitate chloroplast capture, a widely reported cytoplasmic introgression phenomenon among angiosperms, which offers only a tentative, unvalidated mechanistic hypothesis for the topological discordance recovered in the plastid phylogeny of the present study (Yi et al., 2015; Casazza et al., 2012; Cristina Acosta and Premoli, 2010). Introgressive hybridization mediates chloroplast capture, enabling distantly related species to share maternally inherited plastid haplotypes, which could hypothetically generate the blurred, non-monophyletic clades observed in our plastid phylogeny. This represents one speculative pathway that might partially explain why quite a few sections in *Primula* did not belong to the monophyletic group. We emphasize a key limitation of the current analysis: no formal introgression detection or reticulate evolution analyses were performed in this work to directly verify historical hybridization and cytoplasmic transfer signals. All interpretations invoking chloroplast capture to explain non-monophyletic sectional lineages remain purely speculative. Alternative explanations for non-monophyly also include ambiguous morphological sectional circumscriptions and incomplete taxon sampling in our dataset. In summary, our plastid genome-based analysis establishes a robust phylogenetic framework that will serve as a foundational resource for future studies on the evolutionary history, taxonomic classification, and systematics of this genus.

Divergence time analysis indicates that the genus *Primula* originated in the Early Miocene. Within a 13 My window spanning 19.59–6.72 Mya (**Figure 10**), core *Primula* lineages diversified rapidly from a single ancestral lineage into multiple distinct clades. This temporal interval coincides with climatic cooling across the Oligocene–Miocene transition and the intensification of the Asian summer monsoon (Deng et al., 2019; Li et al., 2021; Su et al., 2019). Overall, most extant species of *Primula* initiated their speciation after 5.3 Mya, the onset of the Pliocene, supporting the inference that *Primula* constitutes a typical rapidly evolving genus. Our chronological framework further suggests that the early ancestral lineages of *Primula* were eliminated during the Cretaceous–Paleogene mass extinction event (Collinson and Crane, 1978), and all extant taxa descend from a single surviving ancestral lineage.

Notably, we did not implement formal diversification rate analyses in the present study, so dynamic shifts in speciation rates cannot be statistically validated, and our inferences linking lineage diversification to geological and climatic events remain tentative. Published geological records indicate that the Himalayas had reached near-modern elevations by 17–14 Mya, and persistent orogenic activity generated complex topographies and heterogeneous habitats across the region. Stepwise rapid uplift of the Qinghai-Tibet Plateau (QTP) by the late Miocene rearranged atmospheric circulation and reshaped temperature and precipitation regimes in the Hengduan Mountains, creating a wide array of niches for the evolutionary diversification of *Primula* (Ding et al., 2020; Su et al., 2019). Continuous orogenesis since 14 Mya (Kapp and Decelles, 2019), coupled with enhanced Asian summer monsoon circulation across the Himalaya–Hengduan Mountains, has been repeatedly proposed as a major driver of sustained lineage divergence within *Primula* (Farnsworth et al., 2019; Li et al., 2021; Spicer et al., 2021). In addition, geographic isolation generated by ongoing environmental transformations further facilitated species-level differentiation in this genus (Jiaxin, 2020). Collectively, these intertwined geological and climatic processes promoted parallel speciation within the three major clades recovered in our phylogeny.

Our divergence time estimates are solely based on plastid sequences. Future research integrating nuclear genomic data will help corroborate our chronological estimates, and dedicated rate-shift analyses could further quantify how Miocene climatic and orogenic events shaped the evolutionary dynamics of *Primula*. The time-calibrated phylogeny reconstructed here provides a fundamental baseline for subsequent investigations into the evolutionary history of this genus.

### 4.3 Ancestral character state reconstructions

Ancestral inflorescence reconstruction in *Primula* indicates that the umbel is supported as the ancestral inflorescence type, generating the tentative hypothesis that the highly condensed umbellate structure was already established early in the divergence of the genus. Subsequently, capitulum, spike, and raceme inflorescences evolved independently in some lineages. Inflorescence architecture is not random. It is constrained by developmental mechanics and genetic regulation, then screened by ecological filters such as pollinator behavior, climate, and reproductive requirements, so only forms that maximize pollination and reproductive success persist (Endress, 2010). In many cases, the latter set of forces exerts the stronger influence (Kirchoff and Claßen-Bockhoff, 2013). Previous studies have shown that the frequencies of racemes and cymes are higher in temperate climates than in tropical climates (Prusinkiewicz et al., 2007). The species of the genus *Primula* are predominantly distributed in temperate and cold-temperate regions of the Northern Hemisphere, with the Qinghai-Tibet Plateau–Hengduan Mountains region serving as the center of diversity (Xie et al., 2023b). This aligns logically with our tentative inference from plastid data that the ancestral trait was the umbel. The independent evolution from umbels to racemes or capitulum observed in this study provides a testable evolutionary hypothesis, which may represent an adaptive response to the “increasing interannual variability” of the plateau monsoon climate since the Miocene (Zhisheng et al., 2001).

Ancestral-state reconstruction shows that a purple corolla is the ancestral condition in *Primula*; yellow, white, and other colors evolved repeatedly and independently during later radiations, yielding the hypothesis of frequent adaptive divergence of corolla color within the genus—probably shaped by pollinator preferences or habitat differences. Flavonoid biosynthesis, particularly the production of anthocyanins, is considered a key innovation enabling land plants to adapt to abiotic stresses such as drought and intense ultraviolet radiation (Davies et al., 2024). The ancestral purple corolla state inferred in this study aligns with the background of the genus *Primula* originating in the Qinghai-Tibet Plateau–Hengduan Mountains region, a high-altitude center of diversity, raising a tentative scenario that anthocyanins may have played a crucial protective role in stress tolerance during the early colonization and adaptation of the genus. Our findings are consistent with the prevalent pattern of convergent evolution in flavonoid pathway evolution (Davies et al., 2024). Key enzymes in the anthocyanin biosynthesis pathway, such as F3H and FNS, have undergone independent duplication and functional differentiation events across different plant lineages (Li et al., 2020; Fu et al., 2023). Therefore, the independent loss of anthocyanins in multiple lineages of *Primula* (resulting in yellow or white flowers) reflects the evolutionary repeatability of this trait in response to novel selective pressures.

It is well established that heterostyly is governed by nuclear supergene complexes rather than chloroplast genes (Barrett and Shore, 2008; Huu et al., 2016). However, whole plastid sequences can generate well-resolved genus-wide phylogenetic backbones, which have become a standard framework for reconstructing evolutionary transitions of floral breeding traits in *Primula*. In previous phylogenomic studies of the genus, robust ancestral state inferences of distyly and homostyly were successfully implemented solely on trees derived from complete chloroplast protein-coding gene datasets, without nuclear phylogenetic support (Zhong et al., 2019; Zhou et al., 2017). In our present work, we did not attempt to link plastid genes to floral developmental pathways; instead, we mapped four key floral morphological traits onto the time-calibrated plastid chronogram to trace their evolutionary trajectories. We acknowledge a critical limitation: our phylogeny is built exclusively on chloroplast data, and cytonuclear discordance is common in rapidly radiating clades. All evolutionary reconstructions presented should be treated as hypothesis-generating inferences rather than definitive evolutionary conclusions, and future research incorporating nuclear loci will help further validate or revise our evolutionary hypotheses. *Primula* is a classic model lineage for investigating the evolution of heterostyly (Gilmartin, 2015; Mast and Conti, 2006). Ancestral-state reconstructions indicate that heterostyly is most likely the ancestral condition in the genus, whereas homostylous species are scattered across terminal branches of the phylogeny, implying multiple independent reversals of floral morph. Over macro-evolutionary timescales, the enforced outcrossing associated with heterostyly continuously injects allochthonous alleles, conferring elevated genetic diversity and enhanced adaptive potential. Approximately 92% of *Primula* species retain distyly; the remainder are either purely homostylous or mixed populations containing both morphs (de Vos et al., 2014; Zhong et al., 2019). As de Vos et al. demonstrated, heterostyly effectively mitigates inbreeding depression and reduces extinction risk (Grant and Kalisz, 2020). In contrast, predominantly selfing lineages suffer from eroded genetic diversity and the accumulation of deleterious mutations; coupled with limited seed-dispersal capacity, this leads to a marked decline in long-term fitness. We therefore hypothesize that the elevated extinction rate of selfing clades constitutes a key selective pressure driving the repeated heterostyly-to-homostyly transitions observed across *Primula*.

The reconstruction recovers a campanulate calyx as the ancestral state, whereas the narrowly campanulate form arose multiple times later in the lineage, demonstrating that calyx morphology is evolutionarily labile and may reflect adaptation to disparate ecological settings or pollination strategies. Phylogenetic studies in the highly diverse genus *Eugenia* (Myrtaceae) have similarly revealed that free calyx lobes represent the ancestral state, while various forms of fused calyces have originated independently on multiple occasions (Giaretta et al., 2019). The study further indicates that such transitions rarely reverse, and their function may be related to enhancing floral display for pollinator attraction. This provides a key reference for understanding calyx evolution in *Primula*. Based on this, the recurrent appearance of the narrowly campanulate calyx in this genus likely also represents a largely irreversible adaptive innovation.

## 5 Conclusions

This study reported that the cp genomes from 9 *Primula* species ranged from 151, 555 (*P. secundiflora*) to 154, 653 bp (*P. gemmifera*) with highly conserved overall genomic structure and composition. Codon types with RSCU values ≥ 1.00 were found in 30 (*P. fangii*) to 34 (*P. purdomii*) of the 9 *Primula* species, and the high-frequency preferred codons predominantly terminate in A/T bases. ENC-plot and neutrality plot analyses further demonstrate that natural selection serves as the dominant driver of chloroplast codon usage bias in *Primula*, whereas mutational pressure exerts only a secondary effect; consistent codon evolutionary patterns were recovered across all sampled taxa. A total of 433 SSRs were identified herein, which can serve as molecular markers for future intraspecific diversity studies in *Primula* plants. The variation of IRs was more conservative than that of LSC and SSC. Four highly variable intergenic spacers (*rps16_1-trnQ*, *psbM-trnD*, *rpl32-trnL*, *ndhF-rpl32*) and two gene regions *(rpl32*, *ycf1*) are proposed as candidate molecular markers for subsequent experimental validation in species identification and genetic diversity analyses of *Primula*. The phylogenetic relationships among *Primula* species were constructed, revealing evolutionary affiliations and taxonomic groupings within the genus. *P. dejuniana*, *P. wawushanica*, and *P. meishanensis* are closely related in Sect. *Petiolares*; P. *secundiflora* and P. *purdomii* in Sect. *Proliferae*; *P. gemmifera*, *P. flava* and *P. fangii* all belong to the Sect. *Aleuritia*. Divergence time estimation revealed that *Primula* initiated rapid radiation in the Early Miocene (~19.59 Mya), with most extant species diversifying after the Pliocene (5.3 Mya), supporting its identity as a rapidly evolving genus. Ancestral-state reconstruction tentatively suggests an evolutionary hypothesis that the genus originated with umbel inflorescences, heterostyly and purple corollas; a campanulate calyx is ancestral. This study enriched the complete chloroplast genome resources of *Primula* species and may supply reference data for developing molecular markers, species identification, and phylogeny of *Primula* species.

## Data Availability

The datasets presented in this study can be found in online repositories. The names of the repository/repositories and accession number(s) can be found in the article/**Supplementary Material**.

## Supplementary material

Supplementary Table 1 List of species and their accession numbers in GenBank included in the phylogenetic analysis.

Supplementary Table 2 Codon usage and relative synonymous codon usage (RSCU) values for protein-coding genes in the 9 *Primula* plastid genomes.

Supplementary Table 3 Simple sequence repeats (SSRs) in the 9 *Primula* plastid genomes.

Supplementary Table 4 The repeats in the nine *Primula plastid* genomes.

Supplementary Table 5 Morphological matrix of *Primula.*

Supplementary Figure 1 Maximum likelihood (ML) tree of *Primula* inferred from concatenated plastid CDS alignments.

Supplementary Figure 2 Bayesian inference (BI) tree of *Primula* inferred from concatenated plastid CDS alignments.

Supplementary Figure 3 Phylogeny and time scale of *Primula* species based on concatenated plastid CDS sequences. Species in black stars indicate the nine Primula species studied here. The red bracketed values represent the posterior probability density of the 95% confidence interval.

Supplementary Figure 4 Ancestral state reconstruction of four morphological traits of *Primula* based on the equal-rate (ER) Mk model. **(a)** calyx; **(b)** corolla color; **(c)** inflorescence; **(d)** style.

Supplementary Figure 5 Ancestral state reconstruction of four morphological traits of *Primula* based on the symmetric rate (SYM) Mk model. **(a)** calyx; **(b)** corolla color; **(c)** inflorescence; **(d)** style.

Supplementary Figure 6 Floral morphology of nine *Primula* species in this study. **(a)** *Primula dejuniana*, photo by Cehong Li, PPBC ID: 6222680; **(b)** *Primula meishanensis*, field photograph taken by Zhixi Fu; **(c)** *Primula wawushanica*, photo by Cehong Li, PPBC ID: 26092649; **(d)** *Primula gemmifera*, photo by Qiang Zhu, PPBC ID: 1647475; **(e)** *Primula secundiflora*, photo by Jun Liu, PPBC ID: 1209437; **(f)** *Primula flava*, photo by Dechang Meng, PPBC ID: 14730372; **(g)** *Primula fangii*, photo by Xinxin Zhu, PPBC ID: 2250689; **(h)** *Primula polyneura*, photo by Renbin Zhu, PPBC ID: 5919436; **(i)** *Primula purdomii*, photo by Xinxin Zhu, PPBC ID: 6238309.
